# An integrative longitudinal resilience curriculum

**DOI:** 10.1111/tct.13054

**Published:** 2019-07-12

**Authors:** Shayna Kulman‐Lipsey, Samantha Yang, Arshia Pedram Javidan, Benjamin Fung, Andrea Levinson, Jordyn Vernon, Leslie Nickell, Joanne Leo

**Affiliations:** ^1^ University of Toronto Toronto Ontario Canada

## Abstract

**Background:**

It is well documented that student well‐being is challenged at medical school and that levels of distress increase as students navigate their training. The Doctor of Medicine (MD) programme at the University of Toronto developed a 4‐year resilience curriculum (RC) to encourage students to reach out for help and equip them with resilience‐building strategies to manage adversities in a demanding academic and clinical programme.

… resilience curriculum (RC) to encourage students to reach out for help and equip them with resilience‐building strategies

**Methods:**

Satisfaction surveys, consisting of statements rated by a five‐point Likert scale and short‐answer questions, were distributed to 518 students; in total, data from four workshops were collected. Two focus groups comprising 12 participants in total were facilitated (*n = *6 per group). A thematic content analysis was conducted for the focus group data; open coding was used for transcriptions via an iterative process and inductive analysis.

**Findings:**

Preliminary quantitative and qualitative data suggest that students valued the curriculum. The main themes generated from the thematic content analysis were the value of the RC, the delivery of the RC, and developing a resilient community.

**Discussion:**

More research must be conducted to assess whether the RC has affected student well‐being and resilience. The sustainability of the curriculum depends on the faculty members that support it; faculty development within the areas of wellness and resilience is imperative.

**Innovation and implications:**

The RC, embedded in the core curriculum and integrated within a medical community, is gaining momentum and is valued by students. Further research will assist in the creation of an innovative tool to assess the impact of the RC on medical students.

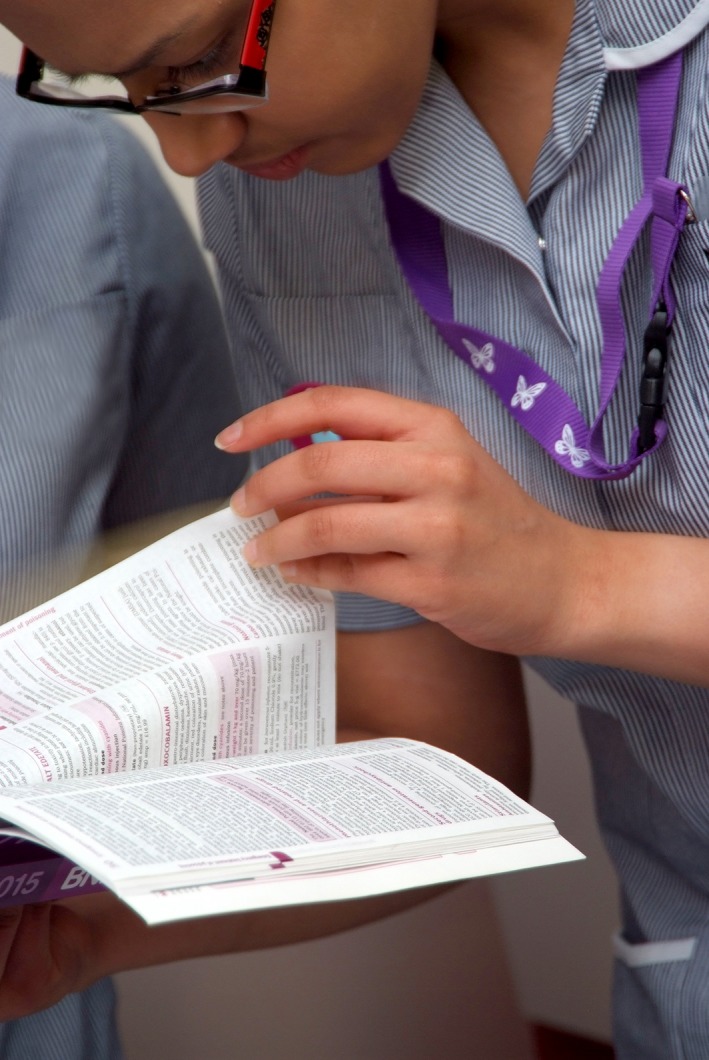

## Introduction

Medical students are at greater risk than the general population of experiencing depression and burnout,^1^ which can lead to medical errors and poorer patient outcomes.^2^ The scope of the problem is extensive; however, there is little in the literature on how to address this pressing issue.^3^

In 2016, the Doctor of Medicine (MD) programme in the Faculty of Medicine at the University of Toronto introduced an innovative resilience curriculum (RC) into the core undergraduate medical curriculum (for the definition of resilience that we adopted, see Box 1).^4^ The uniqueness of the curriculum is that it incorporates the commitment and support of the medical community. The purpose of the RC is to provide students with the tools to enhance their resilience during medical school and to reduce the stigma of reaching out for help.

Box 1 **Working definition of resilience**

Resilience is defined as ‘bouncing back from challenges while growing stronger’^4^

To build this curriculum, the working group asked medical students of all years, ‘what gets in the way of your resilience?’ A list of themes, or barriers to resilience, emerged and were assigned over four class years. Skills to build resilience were identified to address these challenges.^5^, ^6^, ^7^ The themes and skills were supported by evidence from an extensive literature review (Table 1).

**Table 1:** Themes and skill‐building strategies of the resilience curriculum

	Year 1	Year 2	Year 3	Year 4
**Themes of online modules**	Stigma Shame and guilt Imposter syndrome and measuring up Hidden curriculum Upward and downward spirals	Transition to clerkship Values Uncertainty Navigating transitions Expectations and medical error Burnout Compassion fatigue Preventing medical learner suicide	Compassion fatigue versus burn‐out Empathy while setting emotional boundaries Patient dying and death Self‐care and positive coping The importance of debriefing Collaborating with a health care team Fatigue management/ strategies for call and post call Working with feedback	Model for engendering cultural and systemic change within the medical community Dealing with the uncertainty of the residency match
**Skill‐building workshops based on best practices**	Reaching out for help Mindfulness Cognitive reframing Self‐compassion Radical acceptance

… the working group asked medical students of all years, ‘what gets in the way of your resilience?’

**Figure 1:**
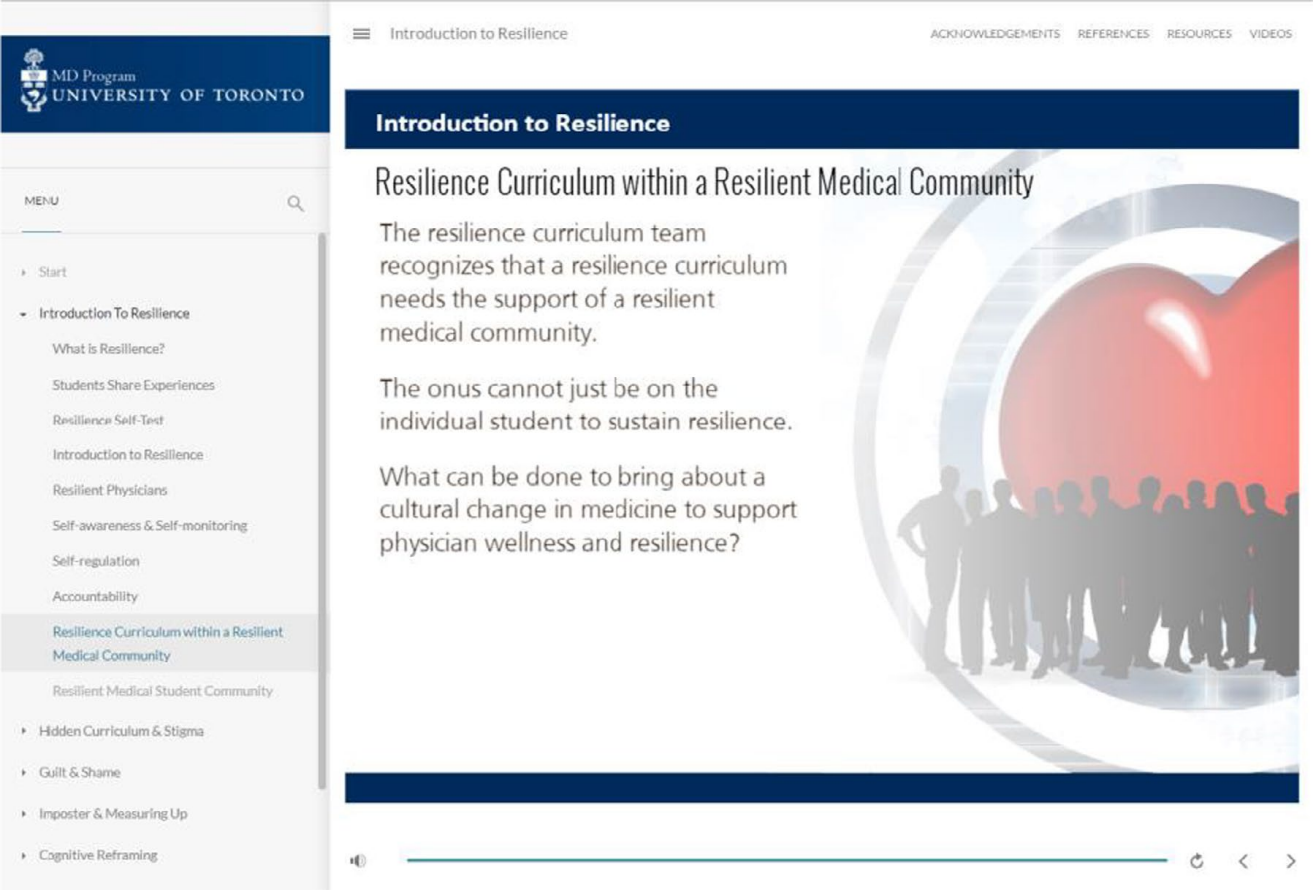
Example of online format of resilience curriculum.

**Figure 2:**
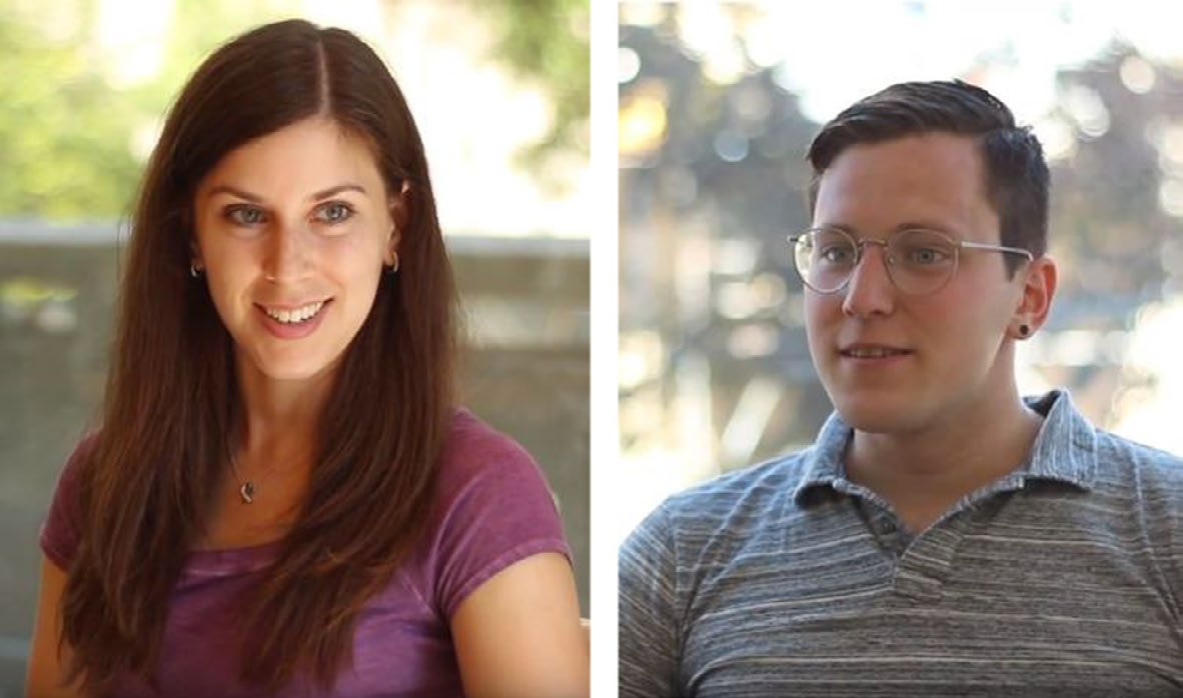
*Monologues in Medicine*: student, trainee, and physician narratives … sharing personal experiences with mental health, wellness and resilience.

The curriculum consists of online modules, compulsory small group workshops and video narratives (*Monologues in Medicine*) of students, residents and physicians sharing personal experiences of mental health, wellness and resilience (Figures 1 and 2).

Students were integral contributors to the development and delivery of the curriculum alongside student affairs staff. Upper‐year students, staff, residents, and physicians in the academic and clinical communities facilitated the workshops.

In response to student input and feedback, there were no graded assessments on the content of the curriculum because of the burden of the pre‐existing assessment schedule.

The RC did not receive any official funding, but costs were integrated into the concurrent Year‐1 and Year‐2 curriculum renewal. To expand the curriculum to all four years, the RC was partnered with the university's Health and Wellness Centre, which provides primary care and psychiatric services for students. With this partnership, the RC was awarded a Medical Psychiatric Alliance Education Innovation Grant in 2017. This grant enabled on‐site psychiatric assessment and treatment for students. Moreover, it provided the opportunity for more robust evaluations and upgrades to the online modules, making them more engaging and user friendly. The RC is able to remain contemporary and sustainable through the leadership of student affairs staff.

In collecting preliminary data, we sought to explore the attitudes of medical students who experienced the RC, with the goal of improving the RC and determining its utility.

## Methods

Participants consisted of Year‐1 and Year‐2 medical students enrolled from autumn 2016 to spring 2017 at the University of Toronto. Each cohort attended two mandatory 2‐hour resilience workshops, involving of 518 students. Satisfaction surveys, involving of statements rated by a five‐point Likert scale and short‐answer questions, were distributed to students following each workshop; in total, data from four workshops were collected. Two months following the last workshop, two focus groups, one for each cohort, were conducted using a semi‐structured interview guide (*n* = 6 students per group; total *n* = 12). Focus group participants were recruited via e‐mail and social media by research delegates. Focus groups were audio‐recorded and transcribed verbatim. Participant data were de‐identified through the use of randomised numeric codes assigned to each participant. Thematic content analysis was used for the workshop short‐answer questions and focus group transcriptions. Open coding was conducted for the focus group transcriptions through an iterative process and inductive analysis.^8^ The codes were reviewed by multiple members of the research team and themes were generated using the constant comparison method.^9^ The framework was verified by an education research scientist and applied deductively to the workshop data.

## Results

A total of 518 students, from both Years 1 and 2 (260 in Year 1 and 258 in Year 2), participated in the RC. Of the 518 students, between 163 and 253 students completed workshop satisfaction surveys, per year, per workshop (Table 2). Quantitative data from the Likert‐scale survey statements demonstrate that across the years and workshops, students were satisfied overall with the quality of the workshops and the extent by which the facilitator involvement encouraged participation, and that workshop objectives were met.

**Table 2:** Quantitative data analysis for satisfaction surveys

Survey statement	‘Workshop objectives as stated were met’	‘Facilitator encouraged participant interaction’	‘Overall satisfaction with the workshop’
**Year 1** **Sept 1**	*m* = 4.63 (*n* = 252, *s* = 0.57)	*m* = 4.85 (*n* = 253, *s* = 0.42)	*m* = 4.54 (*n* = 252, *s* = 0.65)
**Year 1** **Nov 10**	*m* = 4.53 (*n* = 226, *s* = 0.62)	*m* = 4.76 (*n* = 227, *s* = 0.51)	*m* = 4.40 (*n* = 227, *s* = 0.76)
**Year 2** **Oct 30**	*m* = 4.48 (*n* = 163, *s* = 0.59)	*m* = 4.80 (*n* = 163, *s* = 0.51)	*m* = 4.31 (*n* = 163, *s* = 0.70)
**Year 2** **Nov 6**	*m* = 4.49 (*n* = 213, *s* = 0.65)	*m* = 4.82 (*n* = 214, *s* = 0.43)	*m* = 4.42 (*n* = 214, *s* = 0.72)

*m*, mean; *n*, sample size; *s*, standard deviation

For the short answer questions, 78 Year‐1 students and 110 Year‐2 students reported discussion as being valuable; however, 81 Year‐1 students reported not valuing cognitive reframing. A total of 134 Year‐1 students valued mindfulness, whereas this was only valued by 27 Year‐2 students (Table 3).

… three significant themes: the value of the RC; the delivery of the RC; and developing a resilient community

**Table 3:** Quantitative data for short‐answer questions of satisfaction surveys

	Topic	No. of students that value topic	No. of students that did not value topic
**Year 1** ** Sept 1**	Cognitive reframing	39	81
	Self‐compassion	38	13
	Mindfulness	134	21
**Year 1** ** Nov 10**	Resilience strategies	26	5
	Reasons for pursuing medicine	5	7
	Discussion	78	13
**Year 2** ** Oct 30**	Values	41	28
	Uncertainty	20	8
	Transitions	14	5
	Mindfulness	27	17
**Year 2** ** Nov 6**	Resilience strategies	22	7
	Reasons for pursuing medicine	5	7
	Discussion	110	8

The thematic content analysis of the qualitative data emerging from focus groups revealed three significant themes: the value of the RC; the delivery of the RC; and the development of a resilient community.

### Value of the RC

Overall, students found value in the RC. They expressed an appreciation for the workshops and for the *Monologues in Medicine*. They enjoyed learning new strategies, found the skills to be applicable to their lives and perceived some value in all activities.

### Delivery of the RC

Students preferred the delivery of workshops over a lecture‐based format, noting that there was engagement and immersion. The presence of workshop facilitators allowed for effective participation and a safe environment for discussion. Students cited recommendations regarding logistics of delivery such as workshop size, length and scheduled time.

### Developing a resilient community

Students felt that the RC helped support a more resilient community, especially as it normalized their experiences. Other recommendations included consideration of a peer mentorship programme and a campaign to increase broader recruitment for *Monologues in Medicine*. Furthermore, students encouraged facilitators to share more personal experiences and to normalise accessing psychotherapy for students.

## Discussion

Preliminary data have captured the students’ perceived value of the RC via satisfaction surveys and focus groups. Overall, students expressed satisfaction with the curriculum. In particular, many Year‐1 and Year‐2 students, 188 in total, valued the discussion component of the workshops. It is interesting to note, however, that there were some discrepancies among the responses of Year‐1 students with regard to the skills that they found valuable. For example, 81 Year‐1 students reported not valuing cognitive reframing; however, 134 Year‐1 students expressed valuing mindfulness. In contrast, whereas mindfulness was valued among Year‐1 students, only 27 Year‐2 students endorsed mindfulness as being valuable.

The fact that fewer Year‐1 students valued cognitive reframing may be partially attributed to the workshop activity rather than the skill itself. Written feedback from the satisfaction surveys highlighted that some students found the cognitive reframing case example to be too simplistic, and that the activity could have been performed independently rather than in a workshop setting. Furthermore, the decrease in students’ perceived value of mindfulness from Year 1 to Year 2 may be the result of students being saturated by their exposure to mindfulness practice, within the Faculty of Medicine and in the community, as it has become more mainstream in recent years.

Only capturing students’ perceived value of the curriculum is a limitation of our preliminary data collection and analysis. More research must be conducted to see whether the RC has, in fact, had any impact on student well‐being and resilience. Moreover, it would be beneficial to see what it is about the curriculum, and how it has unfolded, that led to the current and future findings. A more robust theory‐based evaluation is in progress to see first how the RC has unfolded from the perspectives of its developers and implementers (i.e. the workshop facilitators). The creation of a validated and standardised resilience assessment tool, as well as a focus group guide that can be shared with other medical schools, are the future directions of the project so it can evaluate the impact of the RC on students for years to come.

Only capturing students’ perceived value of the curriculum is a limitation of our preliminary data

The sustainability of the curriculum depends on the faculty members that support it; therefore, faculty member development within the areas of wellness and resilience is imperative. For this reason, there is another theory‐based evaluation in progress examining faculty member attitudes towards resilience, the RC and practices supporting their own well‐being.

As a student leader of the resilience curriculum said, ‘At University of Toronto, there has been a palpable shift in the kinds of conversations that we are having. Students regularly are able to say to one another and their mentors, “I'm feeling burnt out” or “I think I'm on a downward spiral”. My hope is that teaching these resilience strategies, both formal and informal, will ultimately lead to a new generation of physicians who are serious about their mental health, deliberate about their mental health and are able to maintain their mental health.’^10^

‘At University of Toronto, there has been a palpable shift in the kinds of conversations that we are having …’

## Conclusion

It is well known that the well‐being of medical students deteriorates over the course of medical training. An RC was embedded into the core curriculum of the University of Toronto MD programme. Results from preliminary data indicate that students find the curriculum valuable. Further evaluation is in progress to create a tool to assess the impact of the RC on medical students, and to disseminate these findings to the medical community. The overall aim of this work is to contribute to a cultural shift in medicine that values and responds to the importance of physician wellness and resilience.
